# A flexible approach to identify interaction effects between moderators in meta‐analysis

**DOI:** 10.1002/jrsm.1334

**Published:** 2019-01-09

**Authors:** Xinru Li, Elise Dusseldorp, Jacqueline J. Meulman

**Affiliations:** ^1^ Mathematical Institute Leiden University Leiden The Netherlands; ^2^ Institute of Psychology Leiden University Leiden The Netherlands

**Keywords:** CART, fixed effect, interaction between moderators, meta‐analysis, random effects

## Abstract

In meta‐analytic studies, there are often multiple moderators available (eg, study characteristics). In such cases, traditional meta‐analysis methods often lack sufficient power to investigate interaction effects between moderators, especially high‐order interactions. To overcome this problem, meta‐CART was proposed: an approach that applies classification and regression trees (CART) to identify interactions, and then subgroup meta‐analysis to test the significance of moderator effects. The aim of this study is to improve meta‐CART upon two aspects: 1) to integrate the two steps of the approach into one and 2) to consistently take into account the fixed‐effect or random‐effects assumption in both the the interaction identification and testing process. For fixed effect meta‐CART, weights are applied, and subgroup analysis is adapted. For random effects meta‐CART, a new algorithm has been developed. The performance of the improved meta‐CART was investigated via an extensive simulation study on different types of moderator variables (ie, dichotomous, nominal, ordinal, and continuous variables). The simulation results revealed that the new method can achieve satisfactory performance (power greater than 0.80 and Type I error less than 0.05) if appropriate pruning rule is applied and the number of studies is large enough. The required minimum number of studies ranges from 40 to 120 depending on the complexity and strength of the interaction effects, the within‐study sample size, the type of moderators, and the residual heterogeneity.

## 1 INTRODUCTION

The primary aims of meta‐analysis are to synthesize the estimates of an effect or outcome of interest from multiple studies (ie, effect size) and to assess the consistency of evidence among different studies (ie, heterogeneity test). When study features (ie, moderators) are available, meta‐analysis can be used to assess the influence of the study features on the study outcomes. In recent years, there is a growing need to integrate research findings because of the increasing number of publications. As research questions and data structures are becoming more complex, there are often multiple moderators involved in meta‐analytic data (eg, a study by Michie et al^1^). In such cases, conventional univariate meta‐analytic techniques(2, 3) may not be appropriate. Multivariate meta‐analytic techniques, for example, meta‐regression, are required to assess the influence of multiple moderators on the effect size.

When multiple moderators are available, the effects of moderators may be nonadditive, and the moderators may attenuate or amplify each other's effect. In such situations, interaction effects between moderators occur. Knowledge about interaction effects may provide valuable information. For example, when treatment alternatives consist of several components, the researchers might be interested in questions such as “Which combination of components is most effective?”^4^ Knowledge of effective combinations can be helpful to evaluate existing treatments (eg, by examining whether an effective combination of treatment components is used) and to design new potentially effective treatments (eg, by choosing the effective combinations of treatment components).

Despite the need to investigate multiple moderator variables and the interaction effects between them, most meta‐analytic studies apply univariate moderator analyses only (eg, the study by Huisman et al^5^ and the study by Yang and Raine^6^). And even in studies employing multivariate meta‐analytic techniques, interaction effects were seldom investigated. Possible reasons are the lack of appropriate methods and corresponding software for identifying interaction effects in meta‐analyses. To solve this problem, a new strategy, called meta‐CART,(7, 8) which integrates classification and regression trees^9^ (CART) into meta‐analysis, was proposed. This method can deal with many predictors and represents interactions in a parsimonious tree structure. The results of meta‐CART were promising from a substantial point of view,^7^ that is, the method could produce interpretable and meaningful results for real‐world data. Also, meta‐CART has the potential to be an alternative statistical method for meta‐regression to understand the combined effects of moderators.(10, 11) The results of a previous simulation study showed that regression trees in meta‐CART have better performance than classification trees.^8^ Meta‐CART achieved satisfactory power and recovery rates (ie, greater than or equal to 0.80) with a sufficiently large sample size.

The existing version of meta‐CART has two shortcomings. First, it is a step‐wise procedure. In the first step, the interaction effects are identified by a tree‐based algorithm (ie, CART) using the study effect sizes as outcome variable and the moderators as predictor variables. In the second step, the moderator effects are tested by a subgroup meta‐analysis using the terminal nodes as a new subgrouping variable (with categories referring to the labels of the leaves in which the studies were assigned to by the tree). Second, the fixed‐effect and random‐effects assumptions are not taken into account consistently in meta‐CART. The random‐effects model assumption is considered by the subgroup meta‐analysis in the second step, but not in the splitting procedure of the first step. Furthermore, the fixed‐effect model is assumed in the first step, but not in the testing procedure of the second step.

To overcome these shortcomings, we propose two new strategies, one for the fixed effect model and one for the random effects model, that integrate the two steps of meta‐CART into one. By applying new splitting criteria and a new splitting algorithm, these new strategies of meta‐CART can identify interaction effects and perform the heterogeneity test simultaneously. Furthermore, the model assumption is applied consistently throughout the whole process. The performance of the new strategies of meta‐CART are evaluated via an extensive simulation study with different types of moderators (ie, dichotomous, nominal, ordinal, and continuous). The outline of this paper is as follows. First, we describe shortly the fixed‐effect and random‐effects model in meta‐analysis. Second, we introduce the new strategies of meta‐CART as fixed‐effect meta‐CART and random‐effects meta‐CART with an illustrative example using a real‐world data set. We then evaluate the performance of the two approaches in a simulation study. Finally, we summarize and discuss the results.

## 2 Classification and regression trees

CART is a recursive partitioning method proposed by Breiman et al.^9^ CART includes two types of trees: classification trees (for a categorical outcome variable) and regression trees (for a continuous outcome variable). In this article, we focus on regression trees for meta‐analysis using a continuous outcome variable (ie, the study effect size). A previous study showed that in this framework, regression trees outperformed classification trees.^8^ For a complete introduction for both classification and regression trees, we refer to Merkle and Shaffer.^12^

There are two sequential procedures involved to fit a regression tree: a partitioning procedure that grows a tree to split study cases into more homogeneous subgroups and a pruning procedure that removes spurious splits from the tree to prevent overfitting. The partitioning procedure starts with all cases in one group (ie, the root node). Then the root node is split into two subgroups (ie, offspring nodes) by searching all possible split points across all predictor variables to find the split that induces the highest decrease in heterogeneity (called impurity). The within‐node sum of squares is often used as the impurity for a regression tree. Within a node *j*, the impurity can be written as

(1) $$i(j)=\sum_{(x_{k},d_{k})\in j}{\left(d_{k}-\bar{d}(j)\right)}^{2},$$

where (*x*_*k*_,*d*_*k*_) ∈ *j* denotes the cases (eg, studies in meta‐analysis) that are assigned to node *j* with *x*_*k*_ being the predictor vector (eg, moderators) and *d*_*k*_ being the outcome variable (eg, the study effect size); $\bar{d}(j)$ is the mean of *d*_*k*_ for all cases (*x*_*k*_,*d*_*k*_) that fall into node *j* (see also Breiman et al^9^). The partitioning process can be repeated on the offspring nodes, and each split partitions the parent node into two offspring nodes.

**Figure 1:**
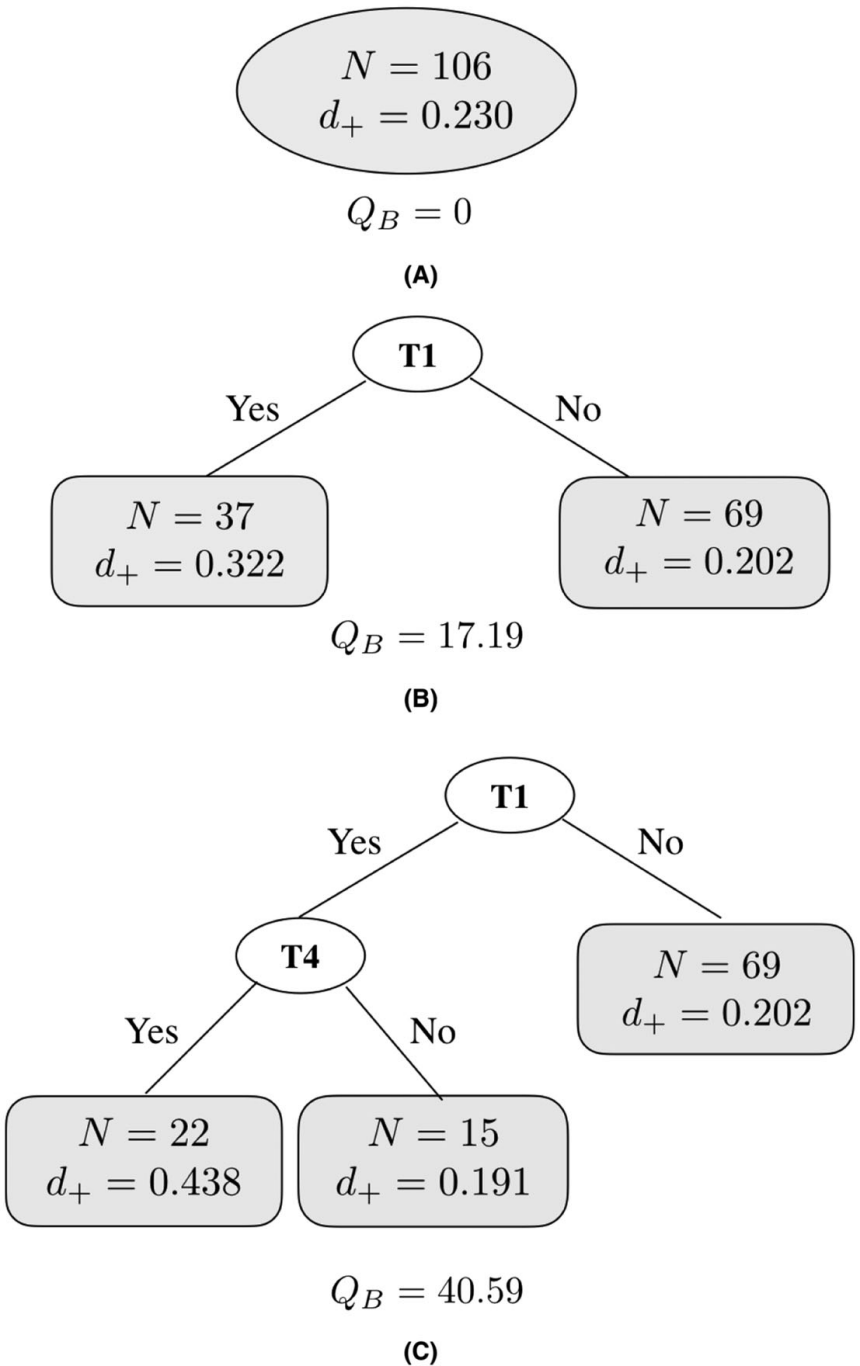
The first three splits of a fixed‐effect (FE) meta‐tree for the studies that applied at least one of the motivation‐enhancing techniques in a study by Michie et al.^1^ T1 and T4 are labels for behavior change techniques “Provide information about behavior‐health link” and “Prompt intention formation,” respectively

For example, in the tree of Figure 1B, a predictor variable “T1” with two values “yes” and “no,” which indicates if the behavior change technique “T1: provide information about behavior‐health link” was applied in a health psychological intervention, is selected as the first splitting variable. If an intervention has applied “T1,” it belongs to the left offspring node. Otherwise, it belongs to the right offspring node. Each of the two offspring nodes can be the candidate of the parent node for the next split.

It is difficult to decide an optimal point to stop the splitting process. Instead, an initial tree is grown as large as possible, and then pruned back to a smaller size by the pruning procedure. To prune a tree, cross‐validation is performed to estimate the sum of squared errors. *
Tenfold cross‐validation is generally recommended.^9^ Tenfold cross‐validation involves splitting up one dataset to 10 folds. To estimate the cross‐validation errors, one fold can be used as the “validation” set, and the left nine folds are used as the “training” set. For each fold used as the validation set, a tree is built using the corresponding training set, and the prediction errors are examined on the validation set.On the basis of the cross‐validation error, there are several pruning rules to select the best size of the tree. To generalize the pruning rules, a pruning parameter *c* can be introduced to select the pruned tree by using the *c*·*S* *E* rule.^13^ The *c*·*S* *E* rule selects the smallest tree with a cross‐validation error that is within the minimum cross‐validation error plus the standard error multiplied by *c*. For standard CART algorithm, Breiman et al suggested using the one‐standard‐error rule to reduce the instability,^9^ which can be regarded as a special case of the *c*·*S* *E* rule when *c* equals 1.

CART is capable of handling high‐dimensional predictor variables of mixed types and excels in dealing with complex interaction effects. It also has the advantage of straightforward interpretability of the analysis results. However, there are two difficulties when applying standard CART in meta‐analysis: (1) the studies are not weighted by their accuracy, and (2) no model assumption is imposed on the algorithm, whereas fixed‐effect and random‐effects assumptions are used in meta‐analysis. We address these two issues and propose solutions in the following sections.

## 3 FIXED‐EFFECT AND RANDOM‐EFFECTS MODEL IN SUBGROUP META‐ANALYSIS

There are two families of statistical models in meta‐analysis: fixed‐effect (FE) models and random‐effects (RE) models.^2^ In this section, we mainly focus on the two models in subgroup analysis, that is, the analysis to evaluate the effect of one categorical moderator in meta‐analysis.

Denote the observed effect size of the *k*th study by *d*_*k*_, FE models assume that

(2) $$d_{k}=\delta +\epsilon_{k},$$

where *δ* denotes the common effect size for all studies, and *ϵ*_*k*_ is the difference between the observed effect size and the true effect size. There is only one source of variance, the within‐study sampling error variance $\sigma_{\epsilon_{k}}^{2}$. In FE meta‐analysis, the summary effect size is computed as the weighted mean with weights $w_{k}=1/\sigma_{\epsilon_{k}}^{2}$:

(3) $$d_{+}=\frac{\sum d_{k}/\sigma_{\epsilon_{k}}^{2}}{\sum 1/\overset{2}{\underset{\epsilon_{k}}{\sigma }}}.$$

In RE models, by contrast, there are two sources of variance: the within‐study sampling error variance and the between‐studies variance. The observed effect size *d*_*k*_ is assumed to be

(4) $$d_{k}=\delta +\tau_{i}+\epsilon_{k},$$

where *δ* is the grand mean of population effect sizes, and *τ*_*i*_ is the deviation of the study's true effect size from *δ*. The summary effect size is computed with weights $w_{k}^{*}=1/(\sigma_{\epsilon_{k}}^{2}+\sigma_{\tau }^{2})$:

(5) $$d_{+}^{*}=\frac{\sum d_{k}/(\sigma_{\epsilon_{k}}^{2}+\sigma_{\tau }^{2})}{\sum 1/(\overset{2}{\underset{\epsilon_{k}}{\sigma }}+\sigma_{\tau }^{2})}.$$

When study features are available in a meta‐analysis, one may perform a subgroup analysis. If a subgroup analysis assumes that the variation of observed effect sizes is only due to the subgroup membership and the within‐study sampling error, the FE model is used, and it allows for no residual heterogeneity. Under these assumptions, the *Q*‐statistic within the *j*th subgroup will be

(6) $$Q_{j}=\sum_{k=1}^{K_{j}}\frac{{\left(d_{jk}-d_{j+}\right)}^{2})}{\sigma_{\epsilon_{jk}}^{2}},$$

where *K*_*j*_ is the number of studies in the *j*th subgroup, *d*_*jk*_ is the observed effect size of the the *k*th study in the *j*th subgroup, and *d*_*j*+_ is the subgroup weighted mean.

The between‐subgroups *Q*‐statistic is given by

(7) $$Q_{B}=\sum_{j=1}^{J}\sum_{k=1}^{K_{j}}\frac{{\left(d_{j+}-d_{++}\right)}^{2}}{\sigma_{\epsilon_{jk}}^{2}},$$

where *J* is the total number of subgroups, and *d*_++_ is the grand weighted mean.

The total weighted sum of squares for all studies is

(8) $$Q_{T}=\sum_{j=1}^{J}\sum_{k=1}^{K}\frac{{\left(d_{jk}-d_{++}\right)}^{2})}{\sigma_{\epsilon_{jk}}^{2}}.$$

There is a simple relationship among *Q*_*j*_, *Q*_*B*_, and *Q*_*T*_ that is analogous to the partitioning of the sum of squares in analysis of variance,

(9) $$Q_{T}=\sum_{j=1}^{J}Q_{j}+Q_{B}.$$

If a subgroup analysis assumes that residual heterogeneity exists, the RE model is used, and it allows for variation unexplained by the subgroup membership and the within‐study sampling error. For subgroup analysis using an RE model, a generally advocated approach is to assume an FE model across subgroups and an RE model within subgroups.^14^ This assumption means that the variation in subgroup means is only explained by the subgroup membership, and the variation in the observed study effect sizes is due to the subgroup membership, the residual heterogeneity between studies, and the within‐study sampling errors. The residual heterogeneity can be estimated separately within subgroups, or a common estimate to all studies can be computed by pooling the within‐subgroup estimates. There are several estimators for the residual heterogeneity available. In this study, we compute the pooled estimate for residual heterogeneity using the DerSimonian and Laird method.^15^ The pooled residual heterogeneity is computed as

(10) $$\sigma_{\tau }^{2}=\frac{\sum_{j=1}^{p}Q_{j}-\sum_{j=1}^{p}df_{j}}{\sum_{j=1}^{p}C_{j}},$$

where *Q*_*j*_ is computed as in (6), *d* *f*_*j*_ equals *K* − 1, and the components *C*_*j*_ using the fixed effects weights, are computed as

(11) $$C_{j}=\sum_{k=1}^{K}w_{jk}-\frac{\sum \overset{2}{\underset{jk}{w}}}{\sum w_{jk}}.$$

The between‐subgroups *Q*‐statistic is given by

(12) $$Q_{B}^{*}=Q_{T}^{*}-\sum_{j=1}^{p}Q_{j}^{*},$$

where

(13) $$Q_{T}^{*}=\sum_{j=1}^{p}\sum_{k=1}^{K}\frac{{\left(d_{jk}-d_{++}^{*}\right)}^{2}}{\sigma_{\epsilon_{jk}}^{2}+\sigma_{\tau }^{2}},$$

and

(14) $$Q_{j}^{*}=\sum_{k=1}^{K}\frac{{\left(d_{jk}-d_{j+}^{*}\right)}^{2}}{\sigma_{\epsilon_{jk}}^{2}+\sigma_{\tau }^{2}}.$$

## 4 FE META‐CART

### 4.1 The algorithm

To solve the two difficulties  when applying standard CART in meta‐analysis, FE meta‐CART applies weights in the CART algorithm and assumes absence of residual heterogeneity when searching for the influential moderators. In FE meta‐CART, we apply the weights used in the FE models in meta‐analysis ( $w_{k}=1/\sigma_{\epsilon_{k}}^{2}$). As a result, the weighted within‐node sum of squares will be equivalent to the *Q*‐statistic within node *j*. Denote the weighted mean of the outcome variable in node *j* as *d*_+_(*j*). It can be shown that

(15) $$d_{+}(j)=\frac{\sum_{(x_{k},d_{k})\in j}(d_{k}\cdot w_{k})}{\sum_{(x_{k},d_{k})\in j}\;(w_{k})}=\frac{\sum_{(x_{k},d_{k})\in j}\;d_{k}/\sigma_{\epsilon_{k}}^{2}}{\sum_{(x_{k},d_{k})\in j}\;1/\sigma_{\epsilon_{k}}^{2}},$$

which is equal to the summary effect size in node *j* under the FE assumption (see Schmidt and Hunter^3^). Also, the impurity function can be computed as

(16) $$i(j)=\sum_{(x_{k},d_{k})\in j}w_{k}{\left(d_{k}-d_{+}(j)\right)}^{2}=\sum_{(x_{k},d_{k})\in j}\frac{{\left(d_{k}-d_{+}(j)\right)}^{2}}{\sigma_{\epsilon_{k}}^{2}},$$

which is equal to the *Q*‐statistic within node *j* as in (6).

When growing an FE meta‐regression tree, the algorithm searches for the moderator and the split point that minimize the sum of *Q*_*j*_ of the offspring nodes. Note that this is equal to the split that maximizes *Q*_*B*_ (see Breiman et al^9^). The splitting process continues until all terminal nodes contain only one or two studies. Then the initial tree will be pruned to a smaller size using cross‐validation to prevent overfitting. For the previous version of meta‐CART, a pruning rule with *c* = 0.5 was generally recommended.^8^ For the new strategies of meta‐CART in this study, we apply two pruning rules with *c* = 0.5 and *c* = 1 and examine their performance. After the pruning process, the final tree gives the corresponding between‐subgroups *Q*_*B*_ and the estimates for summary effect sizes *d*_*j*+_ within each subgroup as the analysis results.

### 4.2 An illustrative example

To illustrate the algorithm, we will use the data from the study by Michie et al^1^ as an example. The complete data consist of 101 studies reporting 122 interventions targeted at physical activity and healthy eating. In this motivating example, we will reanalyze these data focusing on the motivation‐enhancing behavior change techniques (BCTs) that may explain the heterogeneity in the effect sizes of interventions. The interventions that include at least one of the motivation‐enhancing BCTs were selected (*N* = 106). The details about the motivation‐enhancing BCTs can be found in Table 1.

**Table 1:** Overview of the motivation‐enhancing behavior change techniques. The last column displays the number (#) of studies that applied a technique in a study by Michie et al^1^

Technique	Definition	#
1. Provide information about behavior‐health link	General information about behavior risk, for example,	37
	susceptibility to poor health outcomes or
	mortality risk in relation to the behavior	
2. Provide information on consequences	Information about the benefits and costs of action	64
	or inaction, focusing on what will happen if the
	person does or does not perform the behavior	
3. Provide information about other's approval	Information about what others think about the	0
	person's behavior and whether others will
	approve or disapprove of any proposed behavior change	
4. Prompt intention formation	Encouraging the person to decide to act or set	74
	a general goal, for example, to make a behavior
	resolution, such as "I will take more exercise next week"	
5. Motivational interviewing	Prompting the person to provide self‐motivating	17
	statements and evaluations of their own behavior
	to minimize resistance to change	

To identify influential BCTs and the interaction effects between them, FE meta‐CART starts with a root node including all selected studies (Figure 1A). For the first split, the algorithm selects the moderator T1 since it results into the largest between‐subgroups *Q*‐statistic (*Q*_*B*_ = 17.19 among 0.004, 0.10, and 4.35 when choosing the splitting variable as T2, T4, and T5, respectively.) The root node is thereby split into two children nodes (Figure 1B). These two nodes then become the candidates for the parent node for the second split. The algorithm searches through all the combinations of parent node and splitting variable and selects the combination that maximizes the *Q*_*B*_. This splitting process continues until a large tree is grown and all of the terminal nodes only contain one or two studies. Then the large tree is pruned to a smaller size by the cross‐validation procedure, and the final tree is selected as a tree with three terminal nodes shown in Figure 1C.

The final tree represents an interaction effect between the BCTs “T1: provide information about behavior‐health link” and “T4: prompt intention formation.” The main result of this tree is that the combination of “T1” and “T4” results in the highest effect size. More specifically, when “T1” is not applied, the average effect size of the interventions is 0.20 . When “T1” is applied together with “T4,” the interventions have the highest average effect size (0.44). When only “T1” is applied without “T4,” the average effect size is 0.19. The estimated subgroup effect sizes and the between‐subgroups *Q*‐statistic (*Q*_*B*_) are obtained simultaneously as the tree is grown. The final fixed‐effect *Q*_*B*_ equal to 40.59 indicates a significant interaction effect (*P* value <0.001, *d* *f* = 2).

## 5 RE META‐CART

### 5.1 The algorithm

RE meta‐CART takes residual heterogeneity into account and searches for the influential moderators based on the RE between‐subgroups *Q*‐statistic ( $Q_{B}^{*}$) as given in (12). To grow an RE meta‐tree, the algorithm starts with a root node that consists of all studies. In each split of the algorithm, all terminal nodes of the tree obtained from the previous step are considered as candidate parent nodes. To choose a split, two substeps are performed. The first substep is to examine in each candidate parent node the optimal combination of a splitting moderator variable and a split point. By each possible combination of the splitting variable and split point, the candidate parent node can be split into two offspring nodes, and a new branch is formed after the split. For this split, the residual heterogeneity unexplained by the subgroup membership is estimated for the whole tree, and the corresponding $Q_{B}^{*}$ is computed. The first substep then is concluded by selecting across all possible splits the optimal combination that maximizes the $Q_{B}^{*}$. In the second substep, the values of $Q_{B}^{*}$ associated with the optimal combination are compared across all candidate parent nodes, and the node with the highest $Q_{B}^{*}$ will be chosen. After these two substeps, a split is made by splitting the chosen parent node into two offspring nodes (on the basis of the optimal combination of the splitting variable and the split point associated with that parent node).

Same as the splitting process in FE meta‐CART, each new split in RE meta‐CART refreshes the partitioning criterion: the between‐subgroups *Q*‐statistic. However, the RE model implies that the residual heterogeneity $\sigma_{\tau }^{2}$ is reestimated after each split. As a result, a split within one node will globally affect the estimation of $\sigma_{\tau }^{2}$ and the value of $Q_{B}^{*}$. In other words, the within‐subgroup $Q_{j}^{*}$ needs be computed not only for the new offspring nodes, but also for all the other existing terminal nodes in the current tree. Thus, this partitioning method is not fully recursive. Instead, RE meta‐CART applies a sequential partitioning algorithm.

The pruning process of RE meta‐CART is the same as with FE meta‐CART. The initial large tree is pruned back to a smaller size using cross‐validation with the *c*·*S* *E* rule. The associated between‐subgroups $Q_{B}^{*}$, the estimates for residual heterogeneity $\sigma_{\tau }^{2}$, and the within‐subgroup summary effect sizes $d_{j+}^{*}$ are obtained as the final tree is selected.

### 5.2 An illustrative example

**Figure 2:**
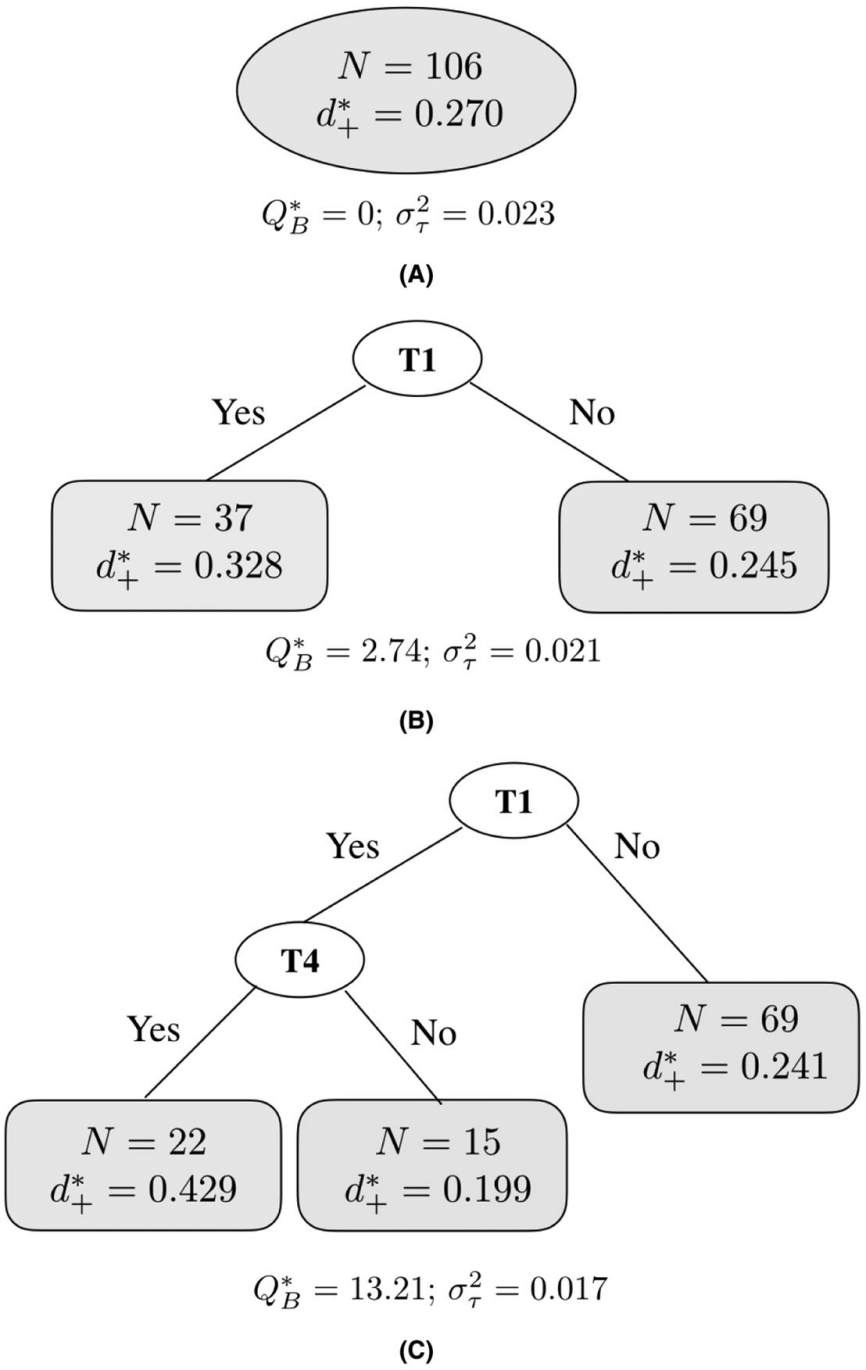
The first three splits of a random effects (RE) meta‐tree for the studies that applied at least one of the motivation‐enhancing techniques in a study by Michie et al.^1^ T1 and T4 are labels for behavior change techniques “Provide information about behavior‐health link” and “Prompt intention formation,” respectively

We will use the same data as in Section 4.2 to illustrate the RE meta‐CART algorithm. To identify the interaction effects using the random effects model, the algorithm starts with a root node including all selected studies (Figure 2A). The first split selects the moderator T1, which results into the largest between‐subgroups *Q*‐statistic ( $Q_{B}^{*}=2.74$ among 0.24, 1.32, and 0.10 when choosing the splitting variable as T2, T4, and T5, respectively.) Then the two children nodes as shown in Figure 2B become the candidates of the parent node for the second split. For the second split, the algorithm searches through all the combinations of parent node and splitting variable and selects the combination that maximizes the $Q_{B}^{*}$. Note that the value of the summary effect size in the unselected node $d_{1+}^{*}$ has been slightly changed from 0.245 to 0.241 after the new split. This change is due to the new estimate for the residual heterogeneity $\sigma_{\tau }^{2}$. Therefore, a new split influences not only the selected parent node but also the unselected node(s). As a result, the sequence of the splits globally influences the estimates for $\sigma_{\tau }^{2}$, $d_{+}^{*}$, and $Q_{B}^{*}$. This sequential partitioning process continues until a large tree is grown and all of the terminal nodes only contain one or two studies †
The exact minimal number of studies in a node is fixed before splitting. We used here a size of two.. After the pruning process, the final tree is selected as a tree with three terminal nodes shown in Figure 2C.

The final tree by RE meta‐CART selects the same moderators as FE meta‐CART in Section 4.2: “T1: provide information about behavior‐health link” and “T4: prompt intention formation.” But under the RE assumption, the estimated summary effect sizes in each subgroup and the between‐subgroups *Q*‐statistic are different from those estimated using FE model. The random effects $Q_{B}^{*}=13.20$ indicated a significant interaction effect (*P* value = 0.001, *d* *f* = 2).

## 6 SIMULATION

### 6.1 Motivation

In the simulation study, we first aim at selecting pruning rules for the new strategies of meta‐CART to control the risk of finding spurious effects (see Section 4.1). Second, we evaluate the performance of FE meta‐CART and RE meta‐CART under various conditions using the selected pruning rules. It is important to note that the simulation study does not aim at comparing FE meta‐CART and RE meta‐CART. The choice of the model assumption should be based on theoretical grounds (also see Section 8.2). The conditions that we consider include observable features of meta‐analytic data sets, such as the number of studies, the within‐study sample sizes, the type of moderators, and the number of moderators, as well as unobservable structures and parameters underlying the data, such as the complexity of the interaction effects, the magnitude of the interaction effect, the correlation between moderators, and the residual heterogeneity. The recovery performance of meta‐CART is measured by the ability of successfully retrieving the true structures underlying the data. In addition, we compare its performance with meta‐regression with true structures specified beforehand, which can be seen as an idealized solution.

**Figure 3:**
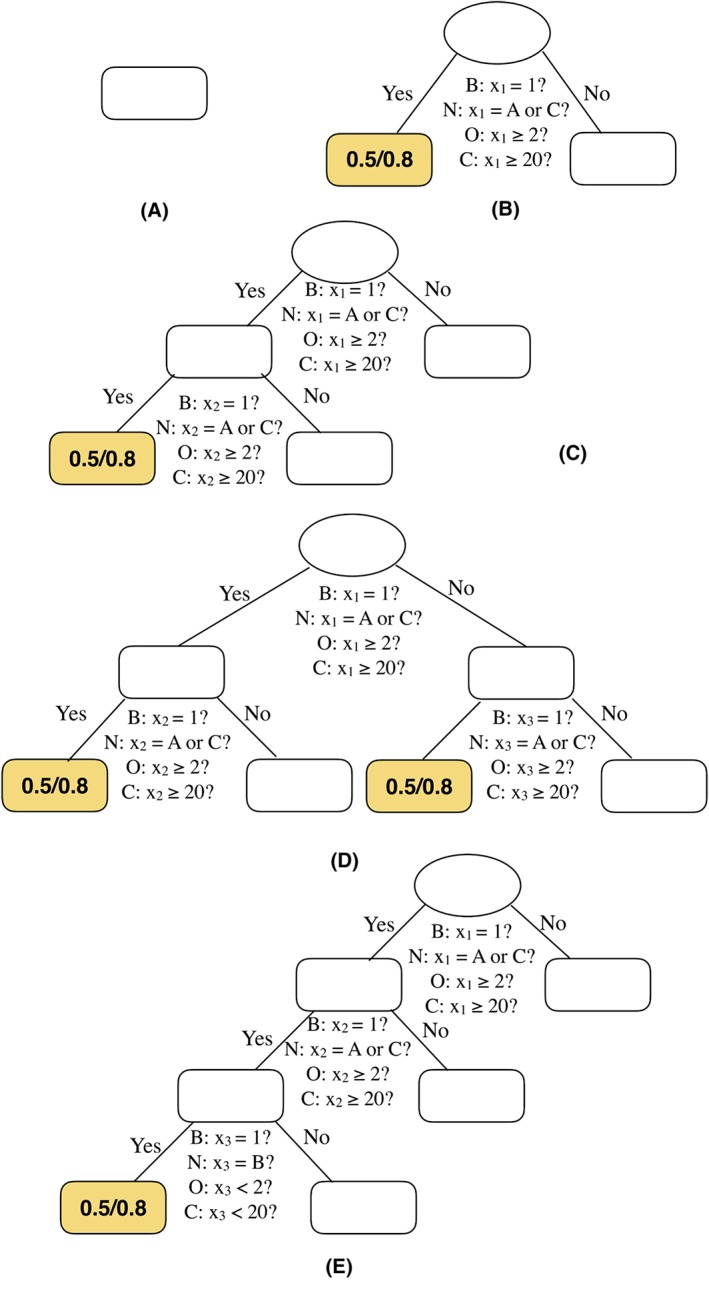
Simulated data sets were generated from five true tree structures: A, to E. The letters B, N, O, and C denote the four types of moderator variables: binary, nominal, ordinal, and continuous, respectively. These tree structures represents a true model including: no moderator effect (model A); only main effect of one moderator (model B); one two‐way interaction (model C); two two‐way interactions (model D); and one three‐way interaction (model E), respectively [Colour figure can be viewed at wileyonlinelibrary.com]

We use a design for the true tree structures that is comparable to the study by Li et al.^8^ Five tree structures with increasing complexity are used as the underlying true model to generate data sets (see Figure 3). Model A was used to assess the probability that meta‐CART falsely identifies (a) moderator effect(s) when there is no moderator in the true model (Type I error). Model B was used to evaluate the ability of meta‐CART to identify the main effect of a single moderator. Models C, D, and E were used to evaluate the extent to which meta‐CART correctly identifies the interaction effects between moderators when interaction effects are present in the true model. In the designed tree model, the treatment is effective only in studies with certain combination(s) of study features. The studies are thereby split on moderators into subgroups. The average effect size in the ineffective subgroups is fixed to be 0, and the average effect size in the effective subgroups was a design factor and is denoted by *δ*_*I*_. The true effect sizes of the studies are generated from a normal distribution with mean equal to the average effect size (ie, 0 for ineffective subgroups and *δ*_*I*_ for effective subgroups) and standard deviation equal to the residual heterogeneity.

### 6.2 Design factors

Artificial data were generated with observed study effect sizes *d*, the within‐study sample size *n*, and potential moderators *x*_1_,…,*x*_*M*_. We used three design factors concerning the moderators. The total number of potential moderators *M* was a design factor with three values: 5, 10, and 20. We generated four different types of moderator variables (*T* *y* *p* *e*): binary, nominal, ordinal, and continuous. In our study, all the ordinal moderators and nominal moderators were generated with three levels (1, 2, 3 for ordinal and A, B, C for nominal). The correlation matrix between the moderators (**R**) was a design factor. Both independent and correlated moderators were generated. To generate uncorrelated moderators we use **R** = **I** as the population correlation matrix. To generate correlated moderators, we used a correlation matrix **R** computed from a real‐world data set by Michie et al.^1^ We first randomly sample *M* moderators from the 26 moderators in the data from Michie et al^1^ and compute the correlation matrix. Then we generate *M* moderators using the computed correlation matrix. The range of correlations varies roughly between −0.40 and 0.40.

In addition to the three design factors concerning the moderators, four other design factors that may influence the effect size *d* were examined: (a) the number of studies (*K*); (b) the average within‐study sample size ( $\bar{n}$); (c) the residual heterogeneity ( $\sigma_{\tau }^{2}$); and (d) the magnitude of the interaction effect (*δ*_*I*_). Three values of *K* were chosen: 40, 80, and 120. Because a previous study showed that meta‐CART applied to data sets with *K* ≤ 20 studies results in poor power rates (less than or equal to 0.30),^8^ therefore we start with *K* = 40. We used the same method as by Viechtbauer^16^ to generate the within‐study sample size *n*_*k*_; the values of *n*_*k*_ were sampled from a normal distribution with an average sample size $\bar{n}$ and standard deviation $\bar{n}/3$. Three levels of the average within‐study sample size $\bar{n}$ were chosen as 40, 80, and 160. The resulting *n*_*k*_ ranged roughly between 15 and 420, which are plausible values encountered in practice. The values of the residual heterogeneity unexplained by the moderators $\sigma_{\tau }^{2}$ were chosen as 0, 0.025, and 0.05. The values of *δ*_*I*_ were chosen as 0.3, 0.4, 0.5, and 0.8, among which 0.5 and 0.8 corresponding to a medium and a large effect size, respectively. 17 A small effect size *δ*_*I*_ = 0.2 was not included in the study, because the previous study showed that meta‐CART failed to have enough power to detect small interaction effect(s).^8^ Thus in total we have *M* × *T* *y* *p* *e* × **R** $\times K\times \bar{n}\times \sigma_{\tau }^{2}\times \delta_{I}=3\times 4\times 2\times 3\times 3\times 3\times 4=2592$ design factors.

### 6.3 Monte Carlo simulation

For each of the five tree structures, 1000 data sets were generated with all possible combinations of design factors (ie, 2592 × 5 × 1000 = 12960000 data sets). To generate continuous moderators, we first generate continuous variables from a multivariate normal distribution with variable means equal to 20, standard deviations equal to 10, and with a correlation matrix as identity matrix (for independent moderators) or a correlation matrix computed as mentioned above (for correlated moderators). Then the generated variables were rounded to the first decimal place to allow for duplicate values. The average number of unique values of the continuous moderators was 37, 71, and 102 for *K* = 40, 80, and 120, respectively. For noncontinuous moderators, we first randomly generate continuous variables from a multivariate normal distribution with a correlation matrix as mentioned above. For binary moderators, the generated continuous variables were dichotomized around their mean. For nominal moderators, the continuous variables were split by the 1/3 quantile and 2/3 quantile of the normal distribution, and the resulting three intervals were randomly labeled by the letters A, B, and C. For ordinal moderators, the continuous variables were split by the 1/3 quantile and 2/3 quantile of the normal distribution and ordered by the intervals that they belonged to. Note that the polytomization attenuates the correlations between the resulting variables. ‡
To prevent attenuation by polytomization, an alternative way could be generating data using polychoric correlations or copulas.


With the given moderators and the tree structure, the average true effect size Δ_*j*_ was computed for each subgroup *j*. For a single study *k* within each subgroup *j*, the true effect size *δ*_*jk*_ was sampled from a normal distribution with mean Δ_*j*_ and variance $\sigma_{\tau }^{2}$. Finally, the observed effect size *d*_*jk*_ was sampled from a noncentral *t*‐distribution, and the corresponding sampling errors $\sigma_{\epsilon }^{2}$ were calculated (see Supporting Material A for detailed information).

### 6.4 The evaluation criteria for success

Three criteria are used to judge the performance of meta‐CART with respect to the true model underlying the data:

Criterion 1.: Meta‐CART falsely detects the presence of moderator effect(s) in the data sets generated from model A (Type I error).
Criterion 2.: Meta‐CART detects the presence of moderator effect(s) in the data sets generated from model B, C, D, or E (power). This criterion evaluates if a nontrivial tree is detected (ie, a pruned tree with at least one split and a significant between‐subgroups *Q*), but does not examine the size of the tree and the correct moderator(s).
Criterion 3.: Meta‐CART successfully selects the moderators used in the true model (recovery of moderator(s)). This criterion examines if the true model is fully recovered with all the true moderators and no spurious moderators are selected.

The computation of these criteria will be specified in Section 6.7.

### 6.5 Comparison with meta‐regression

FE and RE meta‐regression analyses were performed on the datasets generated from nontrivial trees (models B, C, D, and E) with the true moderator effect(s) specified. The analyses results were compared with meta‐CART in terms of recovery of moderators (criterion 3). Note that in this scenario, meta‐regression is expected to result in higher recovery rates, since the true structure is specified in meta‐regression but to be explored by meta‐CART. The goal of this comparison is to evaluate how meta‐CART compares with the optimal performance that meta‐regression can reach in an idealized scenario.

### 6.6 The estimates for subgroup effect sizes

The estimates for subgroup effect sizes were examined in the terminal nodes from the successfully retrieved trees for one cell of the design with medium level of each design factors (ie, Tree complexity = model C, *K* = 80, $\bar{n}=80$, $\sigma_{\tau }^{2}=0.025$, *M* = 10, *δ*_*I*_ = 0.5, **R** = the computed correlation matrix, Variable type = ordinal moderators). Although trees with the first splitting variable as *x*_2_ and the second splitting variable as *x*_1_ are also equivalent to model C, only the trees exactly the same as model C shown in Figure 3 were examined to make the resulting subgroups comparable. For each terminal node in the selected trees, the averaged subgroup effect size estimates were computed, and the proportion that the 95% confidence intervals (CIs) contain the true value were counted.

### 6.7 Analysis

FE meta‐CART and RE meta‐CART were applied to each generated data set using two pruning rules with *c* = 0.5 and *c* = 1.0. The significance of the subgrouping defined by the pruned tree was tested by the between subgroups *Q*‐statistic with *α* = 0.05.

In total, 12960 × 1000 analyses were performed per strategy per pruning rule. Each of the three criteria was evaluated and coded with 0 for “not satisfied” and 1 for “satisfied” for each data set. Subsequently, for each cell of the design, the proportion of “satisfied” solutions was computed per criterion. The resulting proportions were subjected to analyses of variance (ANOVA) with the design factors and their interactions as independent variables. Because of the computation time and the difficulty of interpretation, only four‐way and lower‐order interactions were included as independent variables, and the higher‐order interactions were used as error terms. Partial eta squared^18^ ( ${\hat{\eta }}_{P}^{2}$) was computed for all main effects and interaction terms. For Type I error rate, the pruning parameter *c* was included as a within‐subject design factor, and the generalized eta squared^19^ ([ ${\hat{\eta }}_{G}^{2}$) was computed for all main effects and interaction effect terms. Both FE and RE meta‐CART were compared with meta‐regression on the 9720 × 1000 data sets generated from nontrivial trees. For meta‐regression, criterion 3 is defined as all the true moderator effects being significant (ie, *P* value <0.05). For each cell of the design, the proportion of this criterion being satisfied was computed as the recovery rate. The difference in recovery rates between meta‐CART and meta‐regression within each cell were subjected to ANOVA as mentioned above.

The simulation, the meta‐CART analyses, the meta‐regression analyses, and ANOVA were performed in the R language.^20^ The meta‐CART analyses were performed using the R‐package **metacart**.^21^ The meta‐regression analyses were performed using the R‐package **metafor**.^22^ The R‐codes for the simulation study are available at https://osf.io/mghsz/.

## 7 RESULTS

For the Type I error rate of FE meta‐CART, the ANOVA results reveal that the number of studies (*K*) and the pruning parameter *c* have much stronger influence than the other design factors (see Supporting Material Table S5). For the Type I error rate of RE meta‐CART, the main effect of *K* and the interaction between *K* and *c* have the strongest influence (see Table S6). For both FE meta‐CART and RE meta‐CART, the estimated Type I error rates decrease with increasing *K* and the pruning parameter *c*. Table 2 shows the estimated Type I error rates averaged over the less influential design factors (ie, *T* *y* *p* *e*, **R**, *M*, $\bar{n}$, $\sigma_{\tau }^{2}$, *δ*_*I*_). An average Type I error below 0.05 was chosen to be acceptable in order to control for the risk of finding spurious (interaction) effects. Therefore, the best pruning parameter for FE meta‐CART was selected as *c* = 1 if *K* < 80 and *c* = 0.5 if *K* ≥ 80. And the best pruning parameter for RE meta‐CART was selected as *c* = 1 if *K* < 120 and *c* = 0.5 if *K* = 120. A higher value of *c* indicates more pruning. Thus, for smaller *K*, a higher amount of pruning is needed to control Type I error.

**Table 2:** Type I error rate of meta‐CART, averaged over T y p e, **R**, M, $\bar{n}$, $\sigma_{\tau }^{2}$, δ_I_

		Fixed‐effect meta‐CART			Random‐effects meta‐CART		
model	*c*	*K* = 40	*K* = 80	*K* = 120	*K* = 40	*K* = 80	*K* = 120
A	0.5	**0.071 (0.011)**	0.037 (0.007)	0.023 (0.006)	**0.095 (0.023)**	**0.061 (0.018)**	0.042 (0.014)
	1.0	0.034 (0.009)	0.010 (0.005)	0.004 (0.003)	0.033 (0.011)	0.012 (0.005)	0.005 (0.003)

Type I error rates higher than 0.05 are in boldface. The numbers in parentheses display the standard deviations of the Type I error rates.

For the power rates and the recovery rates of the moderators, ANOVA was employed to analyze the results of meta‐CART using the selected pruning parameters as defined above. For power rates, the ANOVA results on recovery rates reveal that FE meta‐CART and RE meta‐CART are both strongly influenced by the main effects of the number of studies *K*, the magnitude of the interaction effect *δ*_*I*_, the tree complexity (B, C, D, or E), and the residual heterogeneity $\sigma_{\tau }^{2}$ (Tables S7 and S8). In addition, RE meta‐CART is also strongly influenced by the main effects of the average within‐study sample size $\bar{n}$ and the type of moderator variables. Similarly, the recovery rates of FE meta‐CART and RE meta‐CART are both strongly influenced by the main effects of *K*, *δ*_*I*_, the tree complexity, $\sigma_{\tau }^{2}$, and the type of moderator variables (Table S9 and S10). The recovery rates of RE meta‐CART are also strongly influenced by $\bar{n}$. Because the patterns of power and recovery rates are similar and the latter is the more stringent criterion, we focus on the results concerning recovery rates.

**Figure 4:**
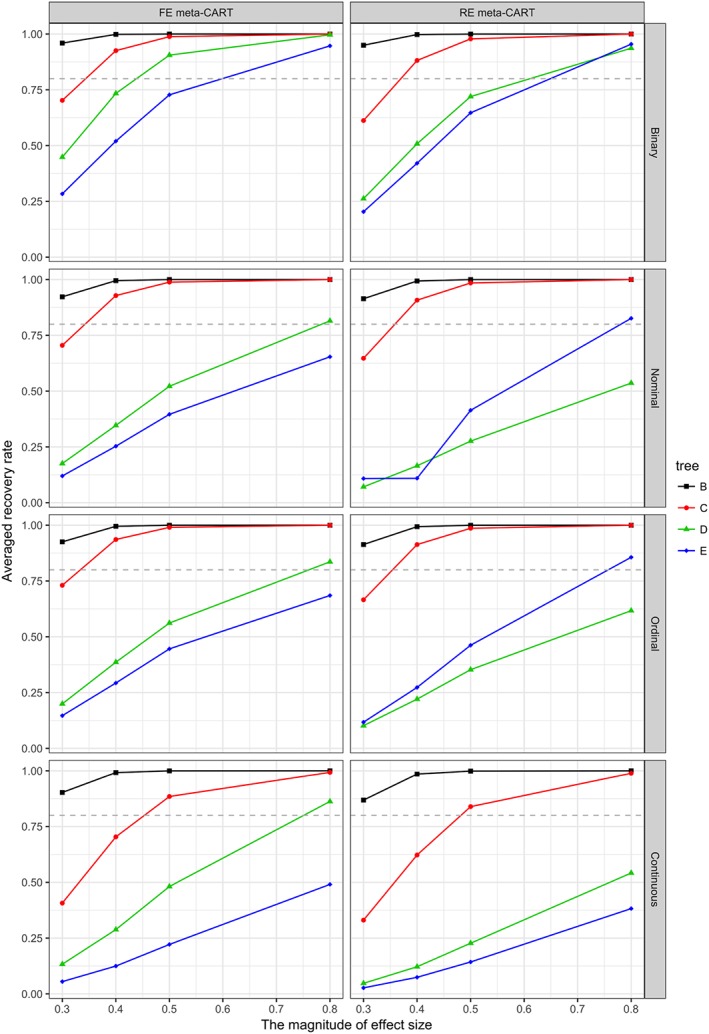
Recovery rates of fixed‐effect (FE) and random‐effects (RE) meta‐CART when K = 120. Separate plots are shown for the types of moderator variables. Separate lines are shown for the tree complexity (models B, C, D, and E). The dashed line indicates a satisfactory recovery rate as 0.80 [Colour figure can be viewed at wileyonlinelibrary.com]

**Figure 5:**
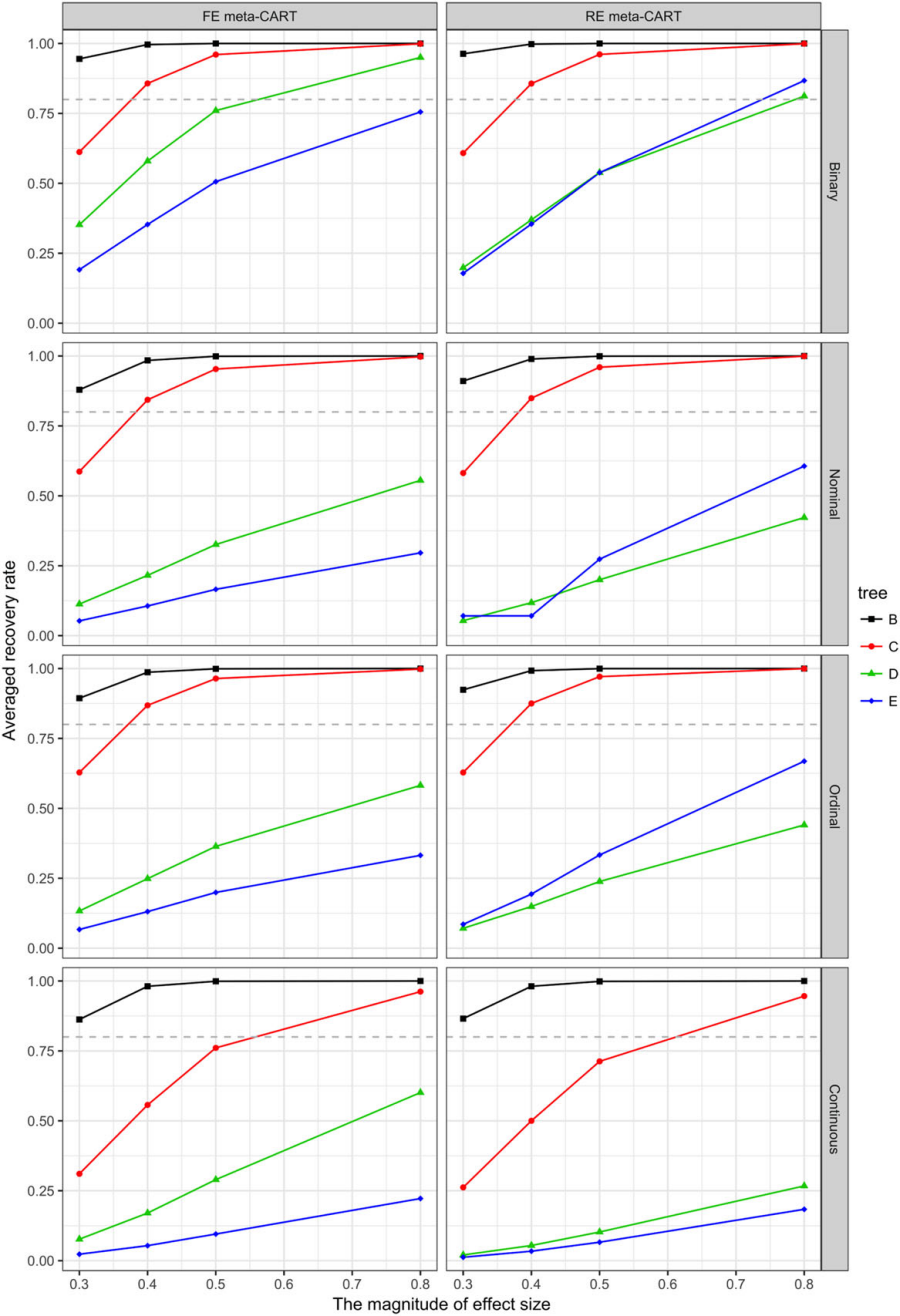
Recovery rates of fixed‐effect (FE) and random‐effects (RE) meta‐CART when K = 80. Separate plots are shown for the types of moderator variables. Separate lines are shown for the tree complexity (models B, C, D, and E). The dashed line indicates a satisfactory recovery rate as 0.80 [Colour figure can be viewed at wileyonlinelibrary.com]

**Figure 6:**
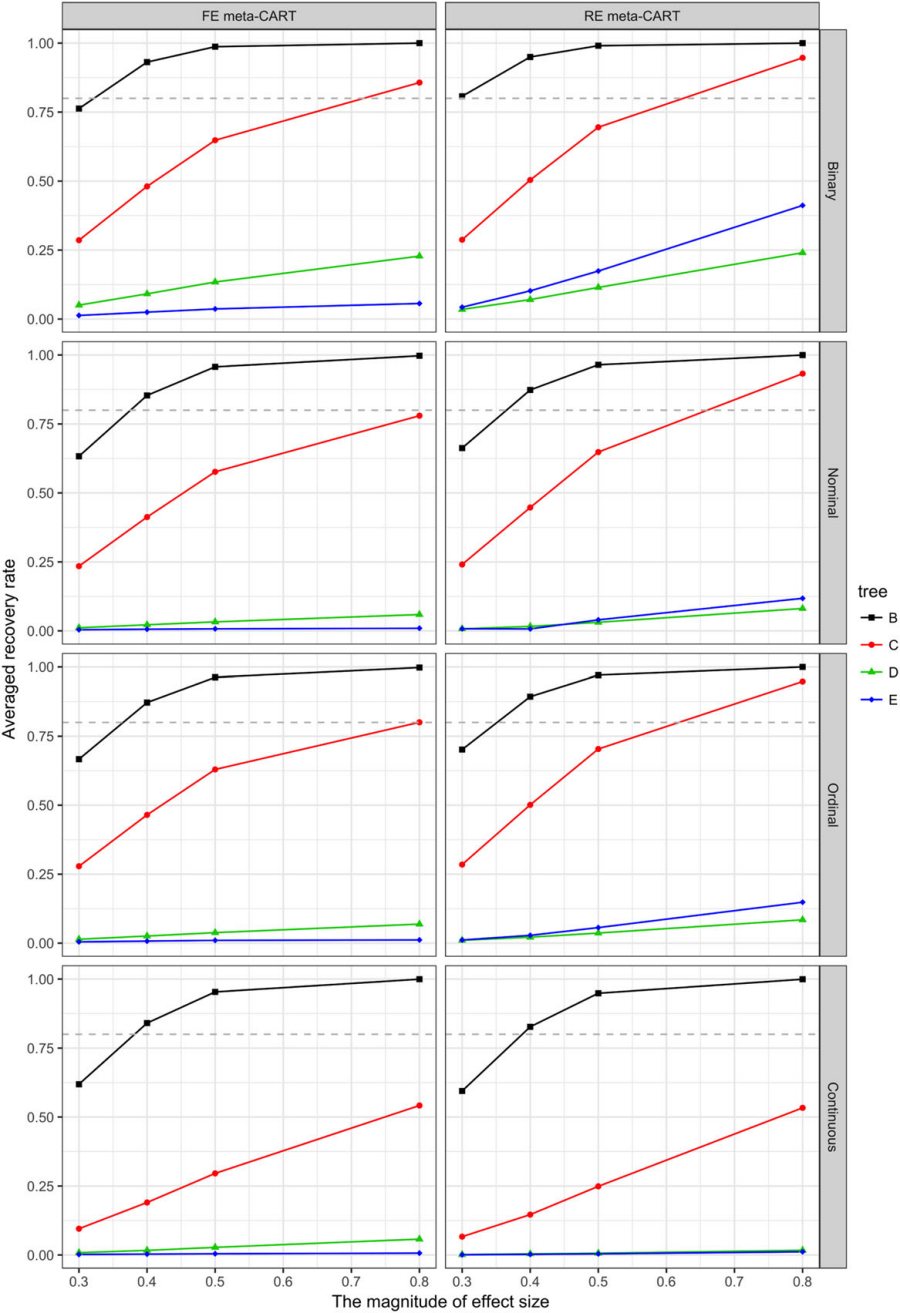
Recovery rates of fixed‐effect (FE) and random‐effects (RE) meta‐CART when K = 40. Separate plots are shown for the types of moderator variables. Separate lines are shown for the tree complexity (models B, C, D, and E). The dashed line indicates a satisfactory recovery rate as 0.80 [Colour figure can be viewed at wileyonlinelibrary.com]

In general, the recovery rates increase with increasing *K*, *δ*_*I*_, and $\bar{n}$, and decrease with increasing $\sigma_{\tau }^{2}$ and tree complexity. Binary moderators have the highest recovery rates, whereas continuous moderators have the lowest recovery rates. The recovery rates for nominal and ordinal moderators are similar. The influence of *K*, *δ*_*I*_, the type of moderator variables, and the tree complexity are shown in Figures 4, 5, and 6. When *K* = 120 (see Figure 4), both FE and RE meta‐CART are able to achieve satisfactory recovery rates (greater than or equal to 0.80) for simple moderator effects (models B and C) in most cases, only with some exceptions when *δ*_*I*_ = 0.3 for noncontinuous moderators or *δ*_*I*_ ≤ 0.4 for continuous moderators. For complex interaction effects (models D and E), meta‐CART is able to achieve satisfactory recovery rates if the interaction effect size is large (*δ*_*I*_ = 0.8) depending on the type of moderators. When the moderators are binary variables, meta‐CART can always achieve satisfactory recovery rates for *δ*_*I*_ ≥ 0.8. When the moderators are nominal or ordinal, FE meta‐CART can achieve satisfactory recovery rates for model D, whereas RE meta‐CART can achieve satisfactory recovery rates for model E. When the moderators are continuous variables, FE meta‐CART can achieve satisfactory recovery rates for model D, but RE meta‐CART fails to achieve recovery rates higher than 0.80. When *K* = 80 (see Figure 5), both FE and RE meta‐CART achieve satisfactory recovery rates for simple moderator effects in most cases, with some exceptions when *δ*_*I*_ = 0.3 for noncontinuous moderators or *δ*_*I*_ ≤ 0.5 for continuous moderators. For complex interaction effects, both FE and RE meta‐CART are able to achieve satisfactory recovery rates for binary moderators if the effect size is large (*δ*_*I*_ = 0.8), but fail to achieve recovery rates higher than 0.80 for nonbinary moderators. When *K* = 40 (see Figure 6), both FE and RE meta‐CART are able to achieve satisfactory recovery rates for simple moderator effects, but fails to achieve recovery rates higher than 0.80 for complex interaction effects. When there is only a univariate moderator effect in the true model (model B), both FE and RE meta‐CART have good performance in most cases. When there is a two‐way interaction (model C), both FE and RE meta‐CART are able to achieve satisfactory recovery rates if the moderators are noncontinuous and the interaction effect size is large (*δ*_*I*_ = 0.8).

For both FE and RE model assumption, the ANOVA results reveal that the difference in recovery rates between meta‐CART and meta‐regression are strongly influenced by the tree complexity (Tables S11 and S12). Table 3 shows the recovery rates and the difference averaged over the less influential design factors (ie, *K*, *T* *y* *p* *e*, **R**, *M*, $\bar{n}$, $\sigma_{\tau }^{2}$, *δ*_*I*_). For simple moderator effects, the recovery rates of meta‐CART are close to meta‐regression with the correct structure specified. For complex interaction effects, the difference is larger.

**Table 3:** Difference in recovery rates between meta‐CART and meta‐regression, averaged over K, T y p e, **R**, M, $\bar{n}$, $\sigma_{\tau }^{2}$, δ_I_

	Fixed effect			Random effects		
Tree	meta‐CART	meta‐regression	difference	meta‐CART	meta‐regression	difference
B	0.94 (0.13)	1.00 (0.02)	−0.05 (0.11)	0.95 (0.12)	0.99 (0.05)	−0.04 (0.08)
C	0.71 (0.32)	0.83 (0.19)	−0.12 (0.20)	0.71 (0.33)	0.71 (0.27)	−0.00 (0.19)
D	0.33 (0.35)	0.72 (0.28)	−0.39 (0.24)	0.22 (0.27)	0.56 (0.34)	−0.34 (0.20)
E	0.21 (0.28)	0.74 (0.27)	−0.53 (0.24)	0.24 (0.31)	0.60 (0.32)	−0.35 (0.26)

The difference is computed as the averaged recovery rates of meta‐CART subtracted by the averaged recovery rates of meta‐regression. The numbers in parentheses display the standard deviations.

From the 1000 data sets generated from the cell of the design described in Section 6.6, 461 trees were selected to examine the estimates for the subgroup effect sizes and the coverage of 95% CIs. Table 4 shows that the averaged estimates are close to the true values in both ineffective subgroups (*δ* = 0) and effective subgroups (*δ* = 0.5). The 95% CIs of FE meta‐CART have lower coverage than the nominated coverage probability, whereas the 95% CIs of RE meta‐CART have coverage close to 0.95 for all three subgroups.

**Table 4:** The estimates for subgroup effect sizes in the successfully retrieved trees (N = 461) from data generated with tree complexity = model C, K = 80, $\bar{n}=80$, $\sigma_{\tau }^{2}=0.025$, M = 10, δ_I_ = 0.5, **R** = the computed correlation matrix, variable type = ordinal moderators

	Fixed‐Effect meta‐CART			Random‐Effects meta‐CART		
	*δ*	averaged $\hat{\delta }$	coverage of 95% CIs	*δ*	averaged $\hat{\delta }$	coverage of 95% CIs
Subgroup 1	0	−0.014 (0.042)	0.835	0	−0.010 (0.053)	0.937
Subgroup 2	0	0.023 (0.058)	0.837	0	0.027 (0.059)	0.933
Subgroup 3	0.5	0.498 (0.037)	0.828	0.5	0.498 (0.044)	0.948

The numbers in parentheses display the standard deviations.

## 8 DISCUSSION

### 8.1 Conclusion, strengths, shortcomings, and remaining issues

This study proposed new strategies for the meta‐CART approach of Dusseldorp et al^7^ and Li et al^8^ as integrated procedures using the FE or RE model and investigated the performance of the new strategies via an extensive simulation study. The simulation results show that the Type I error rates of meta‐CART are mainly influenced by the number of studies included in a meta‐analysis. By varying the pruning rule for different number of studies, the Type I error of meta‐CART is satisfactory (less than or equal to 0.05). The power and recovery rates of meta‐CART mainly depend on the number of studies, the complexity of the true model, the type of moderator variables, the within‐study sample size, the magnitude of interaction effect(s), and the residual heterogeneity. The simulation study used four tree structures with increasing complexity to assess the ability of meta‐CART to retrieve the true model underlying the data set. In general, meta‐CART performed well in retrieving simple models (models with a main effect or one two‐way interaction effect). For more complex models (models with two two‐way interaction effects or a three‐way interaction effect), the power and recovery rates of meta‐CART varied from low (less than or equal to 0.10) to high (greater than or equal to 0.80) depending on the design factors.

The strength of the simulation study is that we extensively examined the influence of both observable design factors (ie, the number of studies, the within‐study sample size, the type of moderators, and the number of moderators) and unobservable design factors (ie, complexity of the interaction effects, the magnitude of the interaction effect, the correlation between moderators, and the residual heterogeneity). These design factors covered various situations that are encountered in practice. By taking into account residual heterogeneity unexplained by the moderators, the simulation study also covers the situations that not all the influential moderators are collected in the data. The results show that the conditions resulting in high performance of meta‐CART and those resulting in low performance are both encountered.

One limitation of the simulation study is that the performance of meta‐CART on mixed types of moderators was not examined, although the algorithm can be applied to data sets with mixed types. The main difficulties of investigating the influence of mixed types of moderators are to define true models to generate artificial data and to define the proportion of each type of the potential moderators in generated data sets. The large number of possible combinations of variable types and proportions within each variable type makes it difficult to extensively examine the performance of meta‐CART on mixed types of moderators. In our study, the simulation results show that the Type I error rates are not largely influenced by the types of moderators, but the power and the recovery rates are strongly influenced by the type of moderators. The recovery rates are highest for binary moderators and lowest for continuous moderators. To roughly assess the performance of meta‐CART on mixed types of moderators, we performed a small simulation study by applying FE meta‐CART to 1000 data sets generated with mixed types of moderators for one combination of the other design factors (*K* = 80, $\bar{n}=80$, **R** = **I**, *M* = 5, $\sigma_{\tau }^{2}=0$). The true model to generate the data consists of a first split with a binary moderator, and two two‐way interactions with an ordinal moderator and a nominal moderator. Given the same combination of other design factors, the estimated power rate of mixed moderators (0.994) is comparable with the estimated power rates of binary, ordinal, and nominal moderators (1.000, 1.000, and 0.998, respectively). The estimated recovery rate of mixed moderators (0.812) is lower than binary moderators (0.991), but higher than ordinal and nominal moderators (0.653 and 0.649, respectively). Thus, it might be plausible to assume that the recovery rates on mixed types of moderators will be in‐between the ones on binary variables and continuous variables. Future study is needed to obtain a solid conclusion about the performance of meta‐CART on mixed types of moderator variables.

Another limitation is that the designed models that were used to generate data did not take linear relationship between effect size and continuous moderators into account. If the effect size is linearly related to continuous moderators, meta‐CART will have difficulties to decide the split points, which is a well‐known disadvantage of tree‐based methods.^23^ One way to solve this problem is to first adjust for the linear relationship (eg, fit a meta‐regression model with main effects of continuous moderators), and then fit a meta‐CART model using the adjusted effect size (ie, the residuals from the first step) as the response variable. Furthermore, for data generated from the designed tree models, meta‐CART has lower recovery rates for continuous moderators than binary, nominal, and ordinal moderators. This might be because the greedy search algorithm of meta‐CART may mistakenly select a local spike when the number of possible split points to be evaluated is large. One possible solution can be using a smooth function to approximate the threshold indicator, for example, the sigmoid smooth function.^24^ It will be interesting to improve the performance of meta‐CART for continuous moderators for both linear and nonlinear relationship in future.

A final limitation is that the simulation study did not examine the coverage of the confidence intervals of the effect size estimates for all combinations of the design factors because of the computation cost and the difficulty to compare subgroups for equivalent trees with different expressions (for an example, see in Supporting Material Figure 7). The analysis results from one cell of the design showed that FE meta‐CART results in too narrow confidence intervals while RE meta‐CART results in confidence intervals with coverage close to the nominated probability. This is because FE meta‐CART ignores the uncertainty introduced by the residual heterogeneity.

One advantage of meta‐CART is that it can deal with multiple moderators and identify interactions between them. In addition, the simulation results show that the performance of meta‐CART is not (largely) influenced by the number of moderators and the correlation between the moderators. Meta‐CART also has the potential to be extended and integrated into other advanced meta‐analytic techniques like multiple group modeling^25^ and meta‐analytic structural equation modeling.^26^ Multiple group modeling is a powerful tool for testing moderators in meta‐analysis, but it can only be used to test for categorical moderators; continuous moderators cannot be assessed with this technique. Meta‐CART can create a subgrouping variable based on continuous moderators, which could be used as a categorical moderator to be tested in a multiple group model. Meta‐analytic structural equation modeling (MASEM) is an increasingly popular technique for synthesizing multivariate correlational research. An extended approach of MASEM by Wilson et al^27^ uses meta‐regression to generate covariate‐adjusted correlation coefficients for input to the synthesized correlation matrix capable of reducing the influence of selected sources of heterogeneity. Since meta‐CART can be used to identify multiple moderators that account for the sources of heterogeneity, it will be interesting to incorporate meta‐CART into MASEM and evaluate the performance in future work. A final advantage is that meta‐CART can keep good control of Type I error (less than or equal to 0.05) by the pruning procedure with cross‐validation. Higgins and Thompson^28^ observed high rates of false‐positive findings from meta‐regression as it is typically practiced. They found that the Type I error rate of FE meta‐regression is unacceptable in the presence of heterogeneity. In addition, the Type I error problems are compounded for both FE and RE meta‐regression when multiple moderators are assessed. Compared with meta‐regression, FE meta‐CART has acceptable Type I error rates even in presence of residual heterogeneity. And the Type I error rates are not largely influenced by the number of moderators for both FE meta‐CART and RE meta‐CART.

Although meta‐CART has a good control of the risk of spurious findings, caution is needed for the interpretation of the significance test. Because the moderator effects are explored and tested on the same data set, the test based on the between‐subgroups *Q*‐statistic is a pseudo *Q*‐test. The test only gives information when the moderator effects are not significant. But it does not confirm the significance of the moderator effects. The Type I error of meta‐CART is mainly controlled by the pruning procedure rather than the *Q*‐test. Therefore, meta‐CART should be used as a hypothesis generating tool (ie, an exploratory method) rather than a hypothesis testing method. To confirm the generated hypothesis, standard meta‐analytical methods such as meta‐regression and subgroup meta‐analysis can be employed to test the influential moderator (interaction) effects on new data.

An interesting phenomenon is that FE meta‐CART had higher recovery rates for model E than model D, but RE meta‐CART showed the opposite. A possible explanation is the difference between model D and the models A, B, C, and E. In contrast to other tree models, the tree size of model D is sensitive to the first splitting variable that the algorithm chooses. For example, if the algorithm chooses the moderator *x*_2_ instead of *x*_1_ as the first splitting variable, the final tree will end up with six instead of four terminal nodes. An illustration of the two equivalent trees can be found in Supporting Material Figure 7. Since RE meta‐CART employs the sequential partitioning procedure, it could be more sensitive to the order of the chosen splitting variables than FE meta‐CART, which employs a recursive partitioning procedure.

As a recursive partitioning method and a sequential partitioning method respectively, both FE meta‐CART and RE meta‐CART use local optimization procedures. Thus, the algorithm may find a local optimum solution rather than a global optimum. For example, when applying to the illustrative data set (see Section 4.2), FE meta‐CART results in a local optimum solution with “T1: provide information about behavior‐health link” being the first splitting variable and “T4: prompt intention formation” being the second splitting variable. However, if we apply a “look‐ahead” procedure that searches through all possible combinations of two splitting variables on the same data set, the resulting solution will have “T4: prompt intention formation” as the first splitting variable and “T1: provide information about behavior‐health link” as the second splitting variable. It results in a higher FE between‐subgroups *Q*‐statistic (*Q*_*B*_ = 41.78) compared with the FE meta‐CART solution (*Q*_*B*_ = 40.59). To overcome this local optimum problem, one promising improvement of meta‐CART would be to develop a global optimization algorithm. Such an algorithm for both FE and RE models can improve meta‐CART from several aspects: 1) to avoid local optimum solutions, 2) to reduce the sensitivity of RE meta‐CART to the sequence of the partitioning as mentioned above, and 3) to make the two different partitioning procedures of FE meta‐CART and RE meta‐CART more similar. Thus, it will be worthwhile to develop a global optimization method in future work.

In this study, the ordinal and nominal moderators were generated with three levels. Because in meta‐analytic practice most ordinal moderators commonly have three levels such as “low,” “medium,” and “high,” and categorical moderators commonly have two or three levels, we did not examine the performance of meta‐CART on moderators with larger number of levels. If there are moderators with different numbers of levels, the greedy search property of meta‐CART might induce a selection bias towards variables that have more possible split points.^29^ A solution to address this selection bias is to adapt the GUIDE (Generalized, Unbiased Interaction Detection and Estimation) algorithm by Loh^30^ to the framework of meta‐CART.

### 8.2 The guideline for application of meta‐CART

On the basis of the simulation results, the recommended pruning rule (expressed as a c*SE rule) depends on the type of research at hand and the number of studies. A higher value of *c* indicates more pruning. If higher power and recovery rates are more important than strict control of Type I error for a specific research problem, a smaller pruning parameter *c* can be used. For example, researchers may apply meta‐CART with a liberal pruning rule using *c* = 0 or 0.5 to gain more power by risking higher Type I error rates. If a strict control of the Type I error (less than or equal to 0.05) is required, a stricter pruning rule using *c* = 1 can be applied when the number of studies *K* < 80, and *c* = 0.5 when *K* ≥ 80 for FE meta‐CART. For RE meta‐CART, the pruning rule *c* = 1 can be used when *K* < 120 and *c* = 0.5 when *K* ≥ 120. To perform a meta‐CART analysis with satisfactory performance (ie, with power and recovery rates both higher than 0.80), a minimum number of studies *K* = 40 is required to detect main effect or simple interaction effect such as one two‐way interaction, and *K* = 80 is required to detect more complex interaction effects.

The choice of whether to use FE or RE meta‐CART should be based on the assumption of the residual heterogeneity and the research question, but not on the power and the recovery rates. General discussion and guidelines about the choice between FE model and RE model in meta‐analysis can be found in Borenstein et al^31^ and Schmidt et al.^32^ For meta‐CART analysis, heterogeneity is likely to exist when the number of studies is large (ie, *K* ≥ 40). In addition, FE meta‐CART may result in overoptimistic confidence intervals when residual heterogeneity exists. To conclude, RE meta‐CART is generally recommended, unless there is a priori grounding for the fixed effect assumption.

## CONFLICT OF INTEREST

The author reported no conflict of interest.

## Supporting information

Table 5: Five most influential factors in the ANOVA on Type I error rate for FE meta‐CART
Table 6: Five most influential factors in the ANOVA on Type I error rate for RE meta‐CART
Table 7: Ten most influential factors in the ANOVA on power rate for FE meta‐CART
Table 8: Ten most influential factors in the ANOVA on power rate for RE meta‐CART
Table 9: Ten most influential factors in the ANOVA on recovery rate of moderators for FE meta‐CART
Table 10: Ten most influential factors in the ANOVA on recovery rate of moderators for RE meta‐CART
Table 11: Ten most influential factors in the ANOVA on difference in average recovery rates between FE meta‐CART and FE meta‐regression
Table 12: Ten most influential factors in the ANOVA on difference in average recovery rates between RE meta‐CART and RE meta‐regression
Figure 7: Two equivalent expressions for model D. The different number of splits depend on the first splitting variable.
