# Supplementary material for: A flexible approach to identify interaction effects between moderators in meta‐analysis
Source: Res Synth Methods. 2019 Jan 9;10(1):134–52. doi: 10.1002/jrsm.1334 (PMC6590644; doi:10.1002/jrsm.1334)
Supplement: Supplementary file 1 — Table 5: Five most influential factors in the ANOVA on Type I error rate for FE meta‐CART Table 6: Five most influential factors in the ANOVA on Type I error rate for RE meta‐CART Table 7: Ten most influential factors in the ANOVA on power rate for FE meta‐CART Table 8: Ten most influential factors in the ANOVA on power rate for RE meta‐CART Table 9: Ten most influential factors in the ANOVA on recovery rate of moderators for FE meta‐CART Table 10: Ten most influential factors in the ANOVA on recovery rate of moderators for RE meta‐CART Table 11: Ten most influential factors in the ANOVA on difference in average recovery rates between FE meta‐CART and FE meta‐regression Table 12: Ten most influential factors in the ANOVA on difference in average recovery rates between RE meta‐CART and RE meta‐regression Figure 7: Two equivalent expressions for model D. The different number of splits depend on the first splitting variable. [file JRSM-10-134-s001.pdf]

A The non-central t-distribution of SMD effect size

In studies that compare treatment and control groups with respect to some continuous outcome variable, standardized mean difference (SMD) is commonly chosen as the measure of effect size. Several measures are available to compute the SMD, of which the most popular one is Cohen's d.[17] It is computed as the mean difference between the treatment and control group divided by the pooled standard deviation leading to the estimator:

$$d_k = \frac{\overline{Y}_k^T - \overline{Y}_k^C}{S_k}, \tag{17}$$

where $\overline{Y}_k^T$ and $\overline{Y}_k^C$ denote, respectively, the mean of the treatment group and control group of the kth study, for $k = 1, \dots, K$, with K being the total number of studies in the meta-analysis. S_k is the pooled standard deviation of the kth study. This standard deviation is computed as

$$S_k = \sqrt{\frac{(n_k^T - 1)(S_k^T)^2 + (n_k^C - 1)(S_k^C)^2}{n_k^T + n_k^C - 2}}, \tag{18}$$

where n_k^T, n_k^T, S_k^T, and S_k^T are, respectively, the treatment and control group sample sizes and standard deviations of the kth study (also see Cohen[17]).

When sample sizes of the individual studies are not sufficiently large, the SMDs are positively biased. Therefore, Hedges[33] introduced a modified estimator for the SMD with a correction for small sample size. This modified estimator is sometimes referred as Hedges' g,[33] and it is given by

$$g_k = d_k \cdot c(m_k), \tag{19}$$

where $m_k = n_k^T + n_k^C - 2$. $c(m_k)$ only depends on m_k and can be computed by

$$c(m_k) = \frac{\Gamma(m_k/2)}{\sqrt{m_k/2}\,\Gamma((m_k-1)/2)}. \tag{20}$$

The constant $c(m_k)$ is less than unity and approaches unity when m_k is large. It can be closely approximated by

$$c(m_k) \approx 1 - \frac{3}{4m_k - 1}. \tag{21}$$

The SMD is not normally distributed. In fact, it is closely related to a non-central t-distribution

$$c(m_k)^{-1}(\tilde{n_k})^{1/2} g_k \sim t_{m_k}(\delta_k \sqrt{\tilde{n_k}}), \tag{22}$$

where δ_k is the true effect size of the kth study, and $\tilde{n_k} = (n_k^T n_k^C)/(n_k^T + n_k^C)$.

B The effect sizes of the design factors

The partial η^2s of the design factors in the ANOVA are shown in Tables 5-10. Only the ten most influential factors are shown for each analysis.

Table 5: Five most influential factors in the ANOVA on Type I error rate for FE meta-CART

The name of predictor variable	generalized partial η^2
K	0.70
c	0.39
$K \times c$	0.06
M	0.04
σ_τ^2	0.03

Table 6: Five most influential factors in the ANOVA on Type I error rate for RE meta-CART

The name of predictor variable	generalized partial η^2
$K \times c$	0.57
K	0.27
Variable type	0.04
c	0.04
M	0.03

Table 7: Ten most influential factors in the ANOVA on power rate for FE meta-CART

The name of predictor variable	partial η^2
Tree complexity	**0.97**
δ_I	**0.95**
K	**0.92**
σ_τ^2	**0.85**
$\bar{n}$	0.75
Variable type	0.71
Variable type $\times$ Tree complexity	0.60
$K \times \delta_I \times$ Tree complexity	0.52
Variable Types $\times \delta_I \times$ Tree complexity	0.50
$\delta_I \times \sigma_\tau^2$	0.49

Table 8: Ten most influential factors in the ANOVA on power rate for RE meta-CART

The name of predictor variable	partial η^2
Tree complexity	**0.98**
δ_I	**0.96**
K	**0.91**
Variable type	**0.88**
σ_τ^2	**0.86**
$\bar{n}$	**0.82**
$K \times \sigma_\tau^2 \times$ Tree complexity	0.64
Variable type $\times$ Tree complexity	0.64
$\delta_I \times \sigma_\tau^2$	0.44
$\delta_I \times$ Tree complexity	0.43

Table 9: Ten most influential factors in the ANOVA on recovery rate of moderators for FE meta-CART

The name of predictor variable	partial η^2
Tree complexity	**0.99**
K	**0.96**
δ_I	**0.95**
σ_τ^2	**0.88**
Variable type	**0.85**
$\bar{n}$	0.79
M	0.70
Variable type $\times$ Tree complexity	0.70
$\delta_I \times \sigma_\tau^2$	0.63
$K \times$ Tree complexity	0.60

Table 10: Ten most influential factors in the ANOVA on recovery rate of moderators for RE meta-CART

The name of predictor variable	partial η^2
Tree complexity	**0.99**
δ_I	**0.96**
K	**0.94**
Variable type	**0.90**
σ_τ^2	**0.89**
$\bar{n}$	**0.85**
Variable type$\times$ Tree complexity	0.73
M	0.63
$\delta_I \times \sigma_\tau^2$	0.55
$\delta_I \times$ Tree complexity	0.50

Table 11: Ten most influential factors in the ANOVA on difference in average recovery rates between FE meta-CART and FE meta-regression

The name of predictor variable	partial η^2
Tree complexity	**0.96**
K	0.69
σ_τ^2	0.65
Variable type	0.62
$K \times \delta_I$	0.58
M	0.48
$K \times \bar{n}$	0.35
$\bar{n}$	0.19
δ_I	0.18
$\mathbf{R}$	0.01

Table 12: Ten most influential factors in the ANOVA on difference in average recovery rates between RE meta-CART and RE meta-regression

The name of predictor variable	partial η^2
Tree complexity	**0.93**
Variable type	0.76
M	0.31
$K \times \delta_I$	0.30
$K \times \bar{n}$	0.09
K	0.08
δ_I	0.03
$\bar{n}$	0.01
$\mathbf{R}$	0.00
σ_τ^2	0.00

C Two representations for model D

In the simulation study, a model with two two-way interactions (model D) was used to generate data with complex interaction effects. This tree model can be represented in two trees with different number of splits. The number of the splits depends on the first splitting variable.

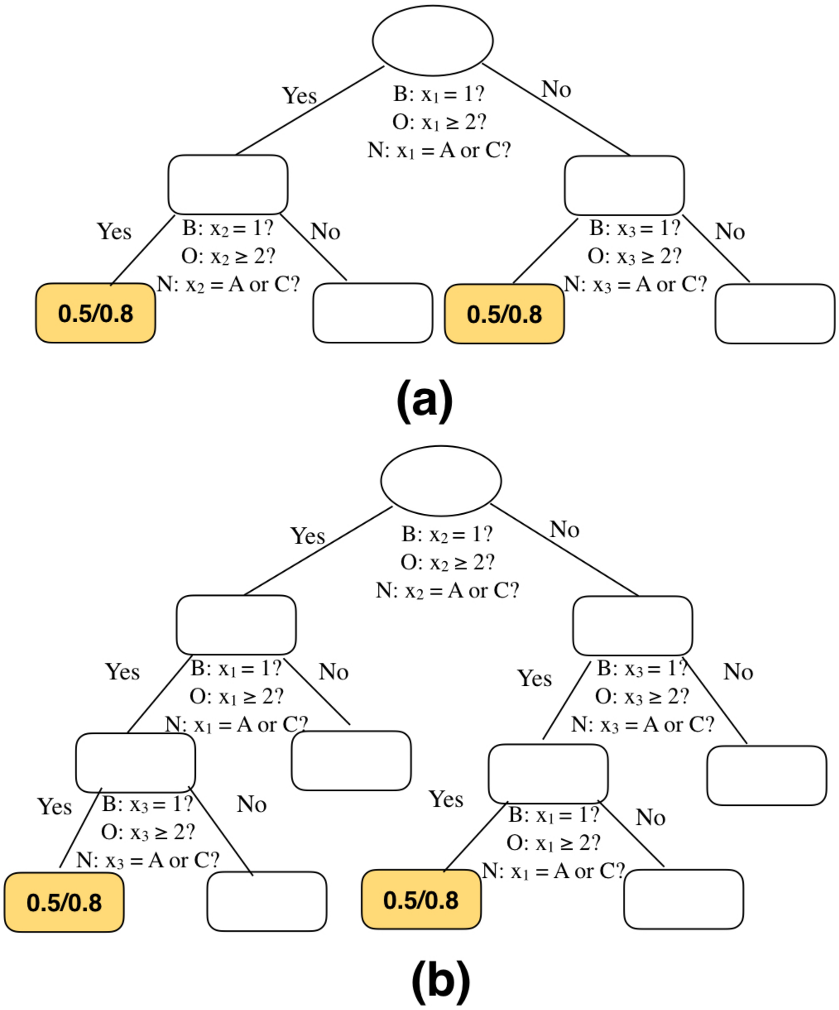

Figure 7: Two equivalent expressions for model D. The different number of splits depend on the first splitting variable.
